# ^212^Pb α-Radioimmunotherapy Targeting CD38 in Multiple Myeloma: A Preclinical Study

**DOI:** 10.2967/jnumed.119.239491

**Published:** 2020-07

**Authors:** Isabelle Quelven, Jacques Monteil, Magali Sage, Amal Saidi, Jérémy Mounier, Audrey Bayout, Julie Garrier, Michel Cogne, Stéphanie Durand-Panteix

**Affiliations:** 1 Nuclear Medicine Department, Limoges University Hospital, Limoges, France; 2 CNRS-UMR7276, INSERM U1262, Contrôle de la Réponse Immune B et Lymphoproliférations, Limoges University, Limoges, France; and; 3 Orano Med SAS, Paris, France

**Keywords:** ^212^Pb, α-radioimmunotherapy, multiple myeloma, CD38

## Abstract

Multiple myeloma (MM) is a plasma cell cancer and represents the second most frequent hematologic malignancy. Despite new treatments and protocols, including high-dose chemotherapy associated with autologous stem cell transplantation, the prognosis of MM patients is still poor. α-radioimmunotherapy (α-RIT) represents an attractive treatment strategy because of the high-linear-energy transfer and short pathlength of α-radiation in tissues, resulting in high tumor cell killing and low toxicity to surrounding tissues. In this study, we investigated the potential of α-RIT with ^212^Pb-daratumumab (anti-hCD38), in both in vitro and in vivo models, as well as an antimouse CD38 antibody using in vivo models. **Methods:** Inhibition of cell proliferation after incubation of the RPMI8226 cell line with an increasing activity (0.185–3.7 kBq/mL) of ^212^Pb-isotypic control or ^212^Pb-daratumumab was evaluated. Biodistribution was performed in vivo by SPECT/CT imaging and after death. Dose-range–finding and acute toxicity studies were conducted. Because daratumumab does not bind the murine CD38, biodistribution and dose-range finding were also determined using an antimurine CD38 antibody. To evaluate the in vivo efficacy of ^212^Pb-daratumumab, mice were engrafted subcutaneously with 5 × 10^6^ RPMI8226 cells. Mice were treated 13 d after engraftment with an intravenous injection of ^212^Pb-daratumumab or control solution. Therapeutic efficacy was monitored by tumor volume measurements and overall survival. **Results:** Significant inhibition of proliferation of the human myeloma RPMI8226 cell line was observed after 3 d of incubation with ^212^Pb-daratumumab, compared with ^212^Pb-isotypic control or cold antibodies. Biodistribution studies showed a specific tumoral accumulation of daratumumab. No toxicity was observed with ^212^Pb-daratumumab up to 370 kBq because of lack of cross-reactivity. Nevertheless, acute toxicity experiments with ^212^Pb-anti-mCD38 established a toxic activity of 277.5 kBq. To remain within realistically safe treatment activities for efficacy studies, mice were treated with 185 kBq or 277.5 kBq of ^212^Pb-daratumumab. Marked tumor growth inhibition compared with controls was observed, with a median survival of 55 d for 277.5 kBq of ^212^Pb-daratumumab instead of 11 d for phosphate-buffered saline. **Conclusion:** These results showed ^212^Pb-daratumumab to have efficacy in xenografted mice, with significant tumor regression and increased survival. This study highlights the potency of α-RIT in MM treatment.

Multiple myeloma (MM) features monoclonal proliferation of plasma cells in bone marrow. Over the last decade, many advances have been made in MM therapy, and the median life expectancy of patients has almost doubled. This improvement was mostly due to the development of proteasome inhibitors, immunomodulatory drugs, histone deacetylase blockers, and, more recently, monoclonal antibodies (mAbs) (daratumumab and elotuzumab) (1). However, the prognosis of myeloma patients remains poor, since remission obtained with such treatments is often followed by relapse. Innovative therapies with a distinct mechanism of action are therefore needed.

Targeted immunotherapy using mAbs has showed efficacy; nevertheless, strategies to enhance mAb efficiency are necessary and were developed in the form of antibody–drug conjugates, immunotoxins, or radiolabeled antibodies. The efficacy of radioimmunotherapy (RIT) in the treatment of non-Hodgkin lymphoma is well established, with there being 2 marketed anti-CD20 mAbs coupled with β-emitters: ^90^Y-ibritumomab tiuxetan (Zevalin; Acrotech Biopharma, LLC) and ^131^I-tositumomab (Bexxar; GlaxoSmithKline) (2). Although ^90^Y-ibritumomab tiuxetan is highly efficient against tumor cells, it carries severe side effects compared with rituximab, notably bone marrow toxicity. Since α-particles have a short pathlength of 50–80 μm (compared with a few millimeters for β-particles), RIT with α-emitting radionuclides is highly attractive and expected to reduce unwanted radiation exposure on normal tissues. The short pathlength of α-particles also explains why α-RIT, by specifically targeting the close environment of each malignant cell, is better suited for micrometastatic and disseminated tumor treatment (3). α-particles produce clustered DNA double-strand breaks and highly reactive hydroxyl radicals when hitting biologic tissues. Their short path range leads to a high-linear-energy transfer of approximately 50–230 keV/μm, compared with 0.1–1.0 keV/μm for β-emitters, making α-emitters 100-fold more cytotoxic. Only a few α-emitters are considered suitable for therapeutic use in cancer patients (4). ^212^Pb represents a good candidate. This radioelement is available at high purity through a ^224^Ra/^212^Pb generator. After administration, ^212^Pb (β-emitter) generates ^212^Bi (α-emitter); thus, ^212^Pb, which has a half-life (10.6 h) relatively convenient for mAb pharmacokinetics, serves as an in vivo α-emitter generator (5). A macrocyclic bifunctional ligand, TCMC (1.4.7.10-tetra-(2-carbamoyl methyl)-cyclododecane), was designed and synthesized to obtain a greater stability in vivo for the chelation of lead isotopes (6). mAb can therefore be easily functionalized with TCMC and then radiolabeled with ^212^Pb. ^212^Pb was first used in a human trial of ^212^Pb-TCMC-trastuzumab in patients with HER2–expressing malignancies (7*,*^8^).

CD38, a 45-kDa stable transmembrane glycoprotein receptor, is expressed at a high epitope density on 95%–100% of malignant plasma cells (9*,*^10^). The CD38 antigen is expressed on activated T cells, monocytes, and NK (natural killer) cells but at much lower levels than found on plasma cells, making it a targeting candidate for RIT using anti-CD38 antibodies. Few RIT studies have evaluated this target potency, and none have evaluated ^212^Pb-anti-CD38 RIT in myeloma. Daratumumab is an antihuman CD38 developed by Janssen and currently used in the clinic (11). We have developed a targeted α-therapy in which the daratumumab antibody is coupled to the α-particle–emitting radioisotope ^212^Pb. The goal of this study was to investigate the potential of ^212^Pb-daratumumab in the treatment of plasma cell malignancies. Biodistribution and toxicity studies were performed on tumor-free and RPMI 8226 myeloma tumor–bearing mice. Considering that ^212^Pb-daratumumab does not cross-react with the murine CD38, biodistribution and toxicity studies were also performed with an antimurine CD38 mAb. The therapeutic efficacy of this treatment was assessed in vitro and in vivo on a subcutaneous xenograft model.

## MATERIALS AND METHODS

### Cell Lines and Mice

The human myeloma RPMI8226 cell line (ATCC) was maintained in supplemented RPMI 1640 medium. C57BL/6 mice (female, 7–10 wk old) were purchased from Janvier Labs. Rag2^−/−^γC^−/−^ mice were kindly provided by Dr. James Di Santo (Institut Pasteur).

### Xenograft Models

Rag2^−/−^γC^−/−^ mice 8–12 wk old received a subcutaneous graft of 5 × 10^6^ RPMI8226 cells in the leg flank (in 100 μL of phosphate-buffered saline [PBS] and Matrigel [Corning] 50/50, v/v). The mice were monitored daily for signs of pain or discomfort. Tumor volume was measured with a caliper 3 times a week. Studies were conducted 13 d after engraftment (tumors between 150 and 400 mm^3^), except for SPECT/CT imaging (20 d/500–800 mm^3^). All in vivo experiments were performed in accordance with animal ethical rules, and all protocols were authorized by the French Ministry of Research according to European Union regulations (APAFIS 15900-201807061621591, version 2).

### mAb Conjugation and Radiolabeling

mAbs were obtained from Janssen: IgG antihuman CD38 (anti-hCD38) (daratumumab), IgG isotypic control, and IgG antimurine CD38 (anti-mCD38, which is not a surrogate of daratumumab as it lacks the in vitro and in vivo functional activity of the anti-hCD38 antibody). Antibodies were conjugated by Macrocyclics with the bifunctional chelating agent TCMC using a proprietary site-specific technique, with approximately 1.3–2 TCMC/mAb.

^212^Pb was produced from ^224^Ra generators provided by Orano Med SAS. Chelation was performed by incubating 1 mg of mAb-TCMC per 37 MBq of ^212^Pb for 15 min at 37°C in 150 mM ammonium acetate, pH 4.5. The labeling yield, assayed by instant thin-layer chromatography, was more than 94%, and specific activity was approximately 37 MBq/mg for the mAbs at the experiment time. The immunoreactivity of ^212^Pb-daratumumab against hCD38 was assessed in vitro, by direct binding assays (12) on RPMI8226 cells, and a dissociation constant of 2.69 ± 1.28 nM was obtained. This value is consistent with known daratumumab affinity (4.36 nM) (13). Furthermore, an immunoreactive fraction of more than 92% was obtained. For SPECT/CT imaging, mAbs were radiolabeled with ^203^Pb. ^203^Pb in 0.5 M HCl was provided by Lantheus Medical Imaging. After the pH of the ^203^Pb solution had been adjusted to 4.5 with 1.5 M ammonium acetate, mAbs were incubated for 15 min with ^203^Pb at 37°C. The labeling yield was more than 98%, and specific activity was approximately 37 MBq/mg.

### Cell Proliferation Analyses

RPMI8226 cells were cultured at 37°C in 96-well plates (25,000 cells in 100 μL/well). Proliferation was assessed in triplicate on days 1–4, under various concentrations of ^212^Pb-mAb (0.185–3.7 kBq/mL) or cold mAb (5–100 ng/mL) using Cell Titer Glo (incubation, 2 h; optical density, 490 nm) (Promega).

### Biodistribution Experiments and SPECT/CT Imaging

Biodistribution experiments were performed on tumor-free or tumor-bearing C57BL/6 or Rag2^−/−^γC^−/−^ mice. ^212^Pb-anti-mCD38, ^212^Pb-daratumumab, or ^212^Pb-isotypic control (1.85 MBq) was injected intravenously. At each time point (2, 6, 12, 18, 24, and 48 h after injection), 2–5 animals per group were euthanized under isoflurane inhalational anesthesia by cervical dislocation. Selected tissues were excised and weighed, and their radioactivity levels were measured with a calibrated γ-counter (Perkin Elmer) (190–290 keV). The uptake of radioactivity in these organs was expressed as percentage injected dose (%ID)/g after correcting for radioactive decay at each time point.

For SPECT/CT imaging, the animals were injected with ^203^Pb-mAbs (18.5 MBq) and imaged under isoflurane inhalational anesthesia (1.8%, 50% air/50% oxygen, 1.4 L/min) at 2, 6, 24, 48, and 96 h after injection with a small-animal SPECT/CT scanner (U-SPECT4/CT; MILabs). Image acquisition lasted 30 min for earlier time-points to 90 min for later time-points. Energy windows were set over the 279-keV peak (±20%), and a general-purpose rat-and-mouse collimator was used (75 holes of 1.5-mm diameter, iterative reconstruction ordered-subsets expectation maximization, and no filter [16 subsets, 6 iterations, and a voxel size of 0.8 mm]). The SPECT resolution with ^203^Pb is estimated to be less than 1 mm. Images were analyzed with PMOD Software (PMOD Technologies).

### Toxicity Studies

Groups of 5–8 tumor-free mice (C57BL/6 or Rag2^−/−^γC^−/−^) received ^212^Pb-daratumumab, ^212^Pb-anti-mCD38 (185–370 kBq), or PBS by intravenous injection. Mice were monitored and weighed daily. At the experimental time point, a complete blood count was performed on an automated hematology analyzer (Cell Dyn; Abbott). Biochemical parameters (aspartate transaminase, alanine transaminase, urea, and creatinine) were measured in blood plasma on an automated biochemistry analyzer (Konelab; Thermo). The percentage of B220-positive cells in blood, bone marrow, and spleen was determined by flow cytometry (AccuriC6; BD). We took advantage of the coexpression of B220 and CD38 (14) to monitor variations in B-lineage using a B220 labeling.

### RIT Experiments

Mice were randomly assigned to experimental groups and received a single intravenous injection of ^212^Pb-daratumumab (185 kBq or 277.5 kBq), ^212^Pb-isotypic control (277.5 kBq), daratumumab (10 μg), daratumumab (16 mg/kg), or PBS. The mice were monitored daily for general appearance. Twice a week, tumor volume was measured and the mice were weighed. They were euthanized when tumors reached a volume of 1 cm^3^, when tumor ulceration occurred, or when the tumor was causing obvious discomfort.

## RESULTS

### ^212^Pb Irradiation–Induced Inhibition of Proliferation of MM Cells

**FIGURE 1.**
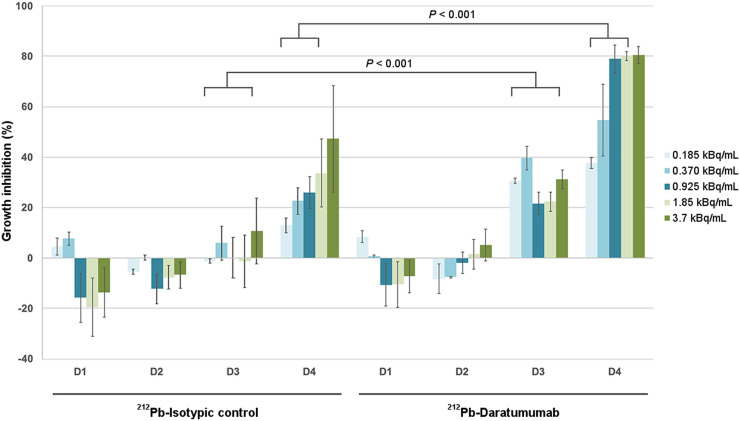
Percentage of growth inhibition for increasing activity of ^212^Pb-mAbs. RPMI8226 cell growth inhibition percentage was calculated, from day 1 to day 4, using untreated cells as controls on same day. Growth inhibition is calculated as [(1 – OD_treatment_/OD_control_) × 100] ± SEM. OD = optical density.

The effect on cell proliferation of increasing activity (0.185–3.7 kBq/mL) of ^212^Pb-daratumumab or ^212^Pb-isotypic control was assessed during 4 d (Fig. 1). Growth inhibition was observed with ^212^Pb-daratumumab from day 3, with a dose-dependent inhibition at day 4 (37.7% ± 2.3% at 0.185 kBq/mL to 80.6% ± 3.4% at 3.7 kBq/mL). This inhibition was significantly higher than ^212^Pb-isotypic control inhibition at all activities tested except for 3.7 kBq/mL. The same experiment with increasing concentrations of the 2 cold mAbs showed no significant effect on growth inhibition (Supplemental Fig. 1; supplemental materials are available at http://jnm.snmjournals.org).

### Biodistribution Experiments and SPECT/CT Imaging

**FIGURE 2.**
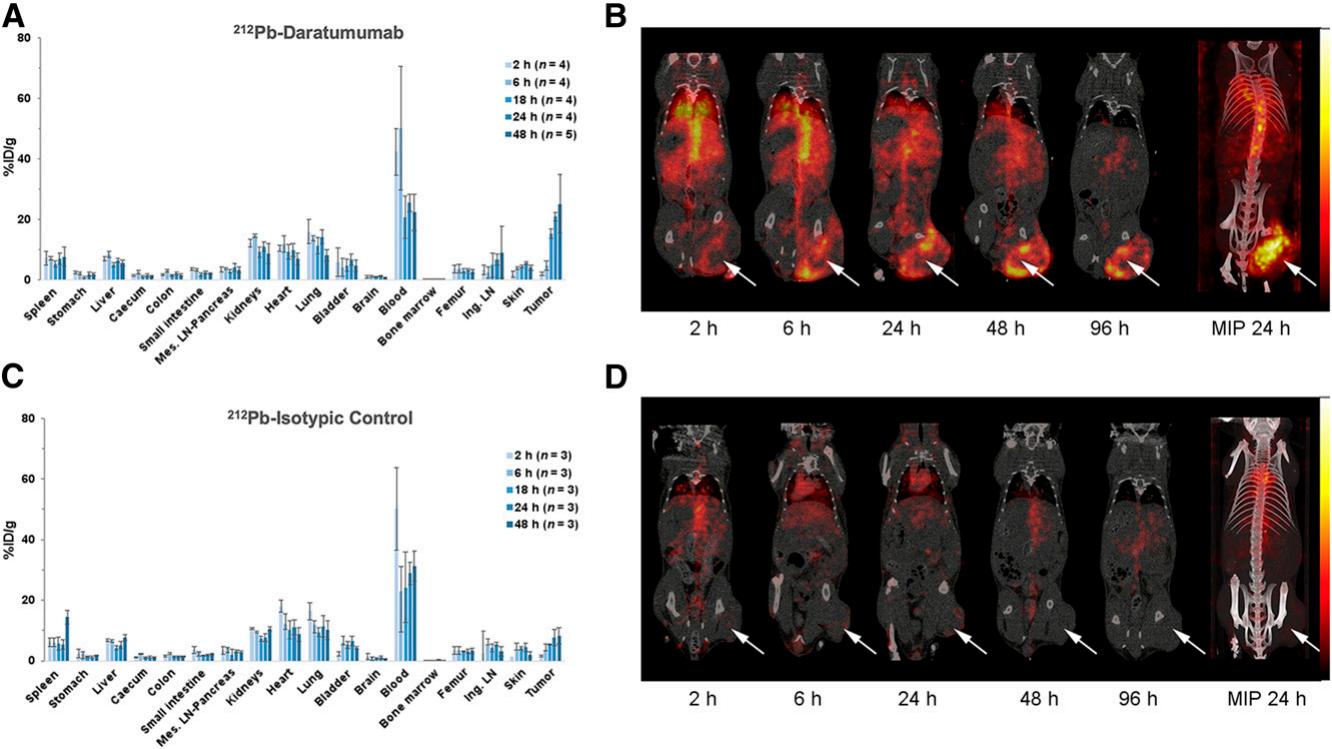
Biodistribution of radiolabeled daratumumab (A and B) and isotypic control (C and D) in mice bearing RPMI8226 xenograft. (A and C) For postmortem biodistribution studies, mice were injected with 185 kBq of ^212^Pb-daratumumab (A) or ^212^Pb-isotypic control (C). Radioactivity in tumor, organs, and blood was expressed as %ID/g. Radioactivity in tumor significantly differs between the 2 mAbs (*P <* 0.001). (B and D) For in vivo imaging studies, 7.4 MBq of ^203^Pb-daratumumab (B) or ^212^Pb-isotypic control (D) were injected. Mice were imaged by small-animal SPECT/CT 2, 6, 24, 48, and 96 h after injection. Tumors are indicated by arrows. Ing. LN = inguinal lymph node; Mes. LN = mesenteric lymph node; MIP = maximum-intensity projection.

Biodistribution of daratumumab was studied in Rag2^−/−^γC^−/−^ tumor-bearing mice to monitor daratumumab-specific accumulation in the human tumor xenograft and was compared with an isotypic control (Fig. 2). After ^212^Pb-daratumumab injection, radioactivity in the tumors increased over time: peak radioactivity was reached after 24 h (20.8 ± 1.4 %ID/g), and this high level was maintained at 48 h after injection. From 18 h, accumulation of ^212^Pb-daratumumab in the tumor was significantly higher than that of ^212^Pb-isotypic control (7.75 ± 2.6 %ID/g at 24 h). In vivo, imaging data confirmed specific tumoral accumulation of daratumumab, with a peripheral tumoral uptake for larger tumors (Figs. 2C and 2D). The accumulation of ^212^Pb-daratumumab and ^212^Pb-isotypic control in all nontumor tissues did not significantly differ.

**FIGURE 3.**
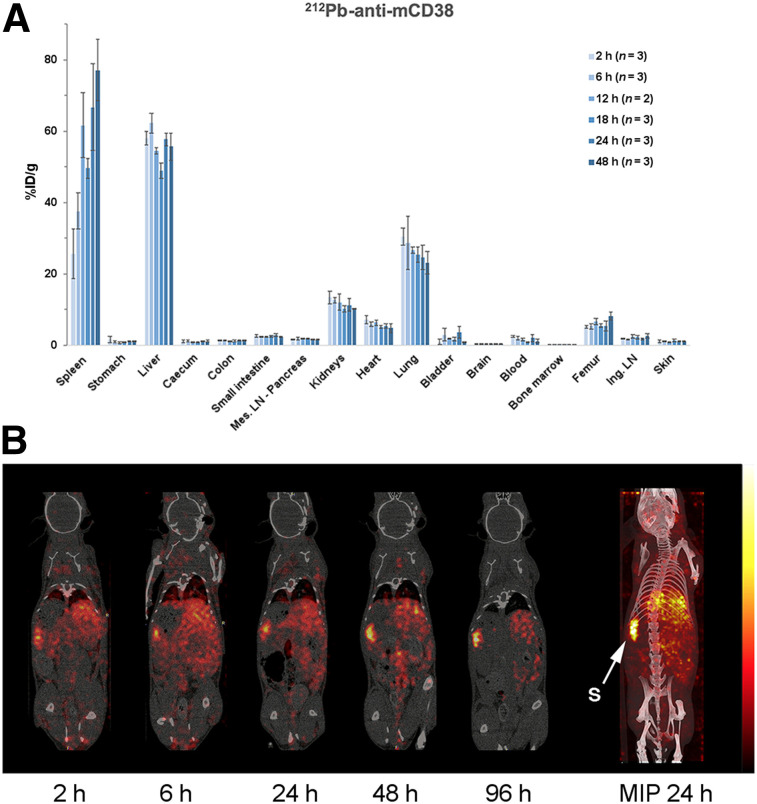
Biodistribution of radiolabeled anti-mCD38 in C57BL/6 healthy mice. (A) For postmortem biodistribution studies, mice were injected with 185 kBq of ^212^Pb-anti-mCD38. Radioactivity was expressed as %ID/g of tissue. (B) For in vivo studies, mice were imaged by small-animal SPECT/CT 2, 6, 24, 48, and 96 h after injection of ^203^Pb-anti-mCD38 (7.4 MBq). Ing. LN = inguinal lymph node; Mes. LN = mesenteric lymph node; MIP = maximum-intensity projection; S = spleen.

Because daratumumab does not bind mCD38, a biodistribution study using an anti-mCD38 antibody was performed on tumor-free mice to estimate antibody accumulation in healthy organs and anticipate potential toxicity issues. Biodistribution studies on C57BL/6 mice showed high accumulation of radioactivity in 3 organs as soon as 2 h after injection: liver (58.1 ± 1.9 %ID/g), spleen (25.7 ± 6.9 %ID/g), and lung (30.5 ± 2.4 %ID/g) (Fig. 3). Uptake was then relatively constant over time in these organs, except for the spleen, in which the radioactivity increased with time (66.8 %ID/g at 24 h). Significant radioactivity was also present in the femur (6%–8%), but radioactivity remained low in the blood (<2% after 6 h). The biodistribution of anti-mCD38 was also examined in Rag2^−/−^γC^−/−^ (Supplemental Fig. 2), and a similar pattern was observed, except for a higher uptake in the spleen (134.6 %ID/g at 24 h) due to the splenic hypotrophy of Rag-deficient mice, as observed on SPECT/CT images.

Compared with ^212^Pb-anti-mCD38, ^212^Pb-daratumumab accumulation in spleen, liver, lung, bone marrow, and femur was lower.

### Dose-Range Finding and Acute Toxicity

#### Anti-hCD38/Daratumumab

Acute toxicity (7 and 21 d) was studied after injection of 185, 277.5, or 370 kBq of ^212^Pb-daratumumab in Rag2^−/−^γC^−/−^ healthy mice. For these 3 activity levels, no effect was observed on survival (Table 1), body weight, blood cell count, or biochemical doses (data not shown), consistent with daratumumab’s lack of binding to mCD38. For that reason, toxicity was thus also studied with anti-mCD38.

**TABLE 1:** Mice Surviving After Acute Toxicity Study

			Surviving mice at endpoint (*n*)	
Mouse	Treatment	Activity (kBq)	7 d	21 d
Rag2^−/−^γC^−/−^	PBS		5/5	5/5
	^212^Pb-daratumumab	185	5/5	5/5
		277.5	5/5	5/5
		370	5/5	5/5
	^212^Pb-anti-mCD38	185	5/5	5/5
		370	4/5*	3/5*
C57BL/6	PBS		5/5	5/5
	^212^Pb-anti-mCD38	185	6/6	6/6
		370	8/8	2/8*

*Some mice did not survive to endpoint.

#### Anti-mCD38

**FIGURE 4.**
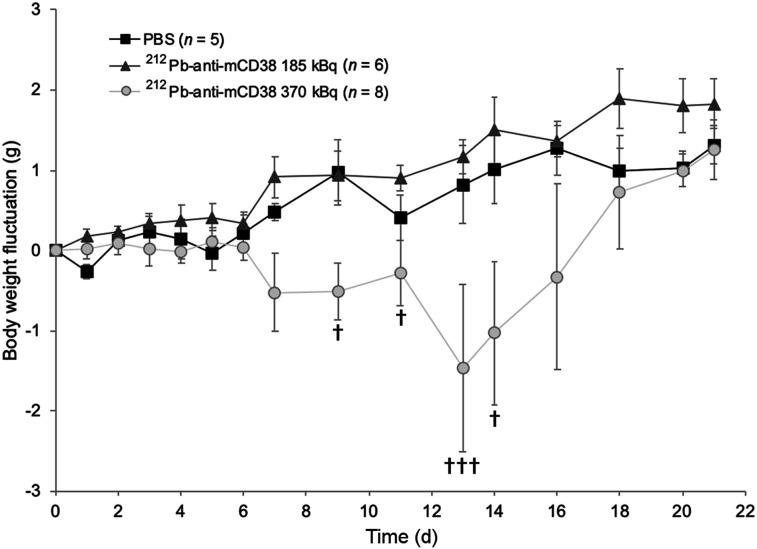
Body weight variations of C57BL/6 mice after acute toxicity study. Mice were injected intravenously with PBS or 185 or 370 kBq of ^212^Pb-anti-mCD38. Variations in body weight relative to day 0 are represented. ^†^Mice euthanized.

The acute toxicity of 185 and 370 kBq of ^212^Pb-anti-mCD38 was studied in C57BL/6 and in Rag2^−/−^γC^−/−^ healthy mice. Animal behavior and body weight were monitored during 21 d. For both strains, a significant body weight loss (*P <* 0.01) was observed after 185 kBq of ^212^Pb-anti-mCD38 compared with PBS (Fig. 4; Supplemental Fig. 3). This loss was dose-dependent and significantly increased with 370 kBq of ^212^Pb-anti-mCD38 (*P <* 0.0001). Survival was not affected after injection of 185 kBq of ^212^Pb-anti-mCD38, whereas 370 kBq of ^212^Pb-anti-mCD38 induced 75% lethality for C57BL/6 and 40% for Rag2^−/−^γC^−/−^ mice at the 21-d time point (Table 1).

**FIGURE 5.**
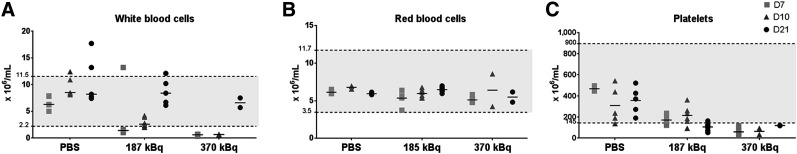
Effect of acute toxicity on blood cell counts. C57BL/6 mice were injected intravenously with PBS or 185 or 370 kBq of ^212^Pb-anti-mCD38. Mice followed over 7 d were euthanized, and blood samples were analyzed. For mice followed over 21 d, blood samples were analyzed on days 10 and 21. Results for white blood cells (A), red blood cells (B), and platelets (C) are represented.

**FIGURE 6.**
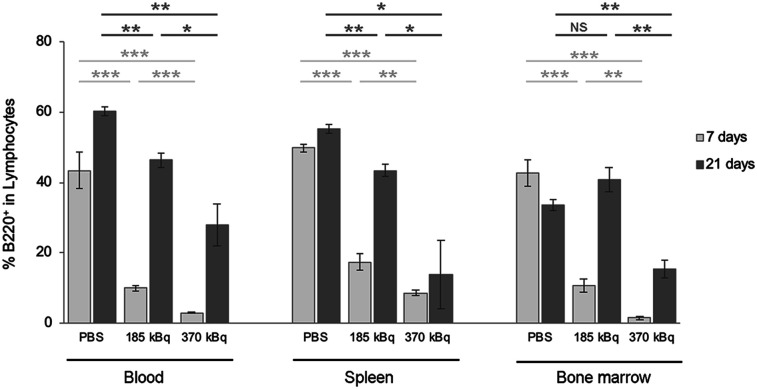
Effect of acute toxicity on B220 cells of lymphoid organs. C57BL/6 mice were injected intravenously with PBS or 185 or 370 kBq of ^212^Pb-anti-mCD38. At endpoint, 7 or 21 d, blood (left), spleen (middle), and bone marrow (right) were excised and analyzed. NS = *P >* 0.05. *P < 0.05. **P < 0.01. ***P < 0.001.

In C57BL/6 mice, we investigated kidney and liver toxicity using biochemical quantification of plasma creatinine, urea (kidney toxicity), aspartate transaminase, and alanine transaminase (liver toxicity). No significant changes in these amounts were observed (Supplemental Fig. 4). Complete blood cell counts revealed hematologic toxicity, with a drop in both leukocyte and platelet counts, whereas red blood cell count was not affected by ^212^Pb-anti-mCD38 injection (Fig. 5). At 7 and 10 d after injection, the leukocyte counts were reduced to the lower limit of the reference ranges for 185 kBq and below the reference ranges for 370 kBq (Fig. 5A). The leukocyte decrease was reversible for both activity levels, and values were back to normal 21 d after injection. In the same way, platelet counts were reduced at as early as 7 d, but no recovery was observed at day 21 (Fig. 5C). Fluorescence-activated cell sorting analyses of the B220 population in blood, spleen, and bone marrow (Fig. 6) confirmed these results and radiation-induced spleen and bone marrow damage.

Acute toxicity manifested at 370 kBq of ^212^Pb-anti-mCD38, and therapeutic activity should thus remain below this dose.

### Efficacy of ^212^Pb-Daratumumab Treatment

**FIGURE 7.**
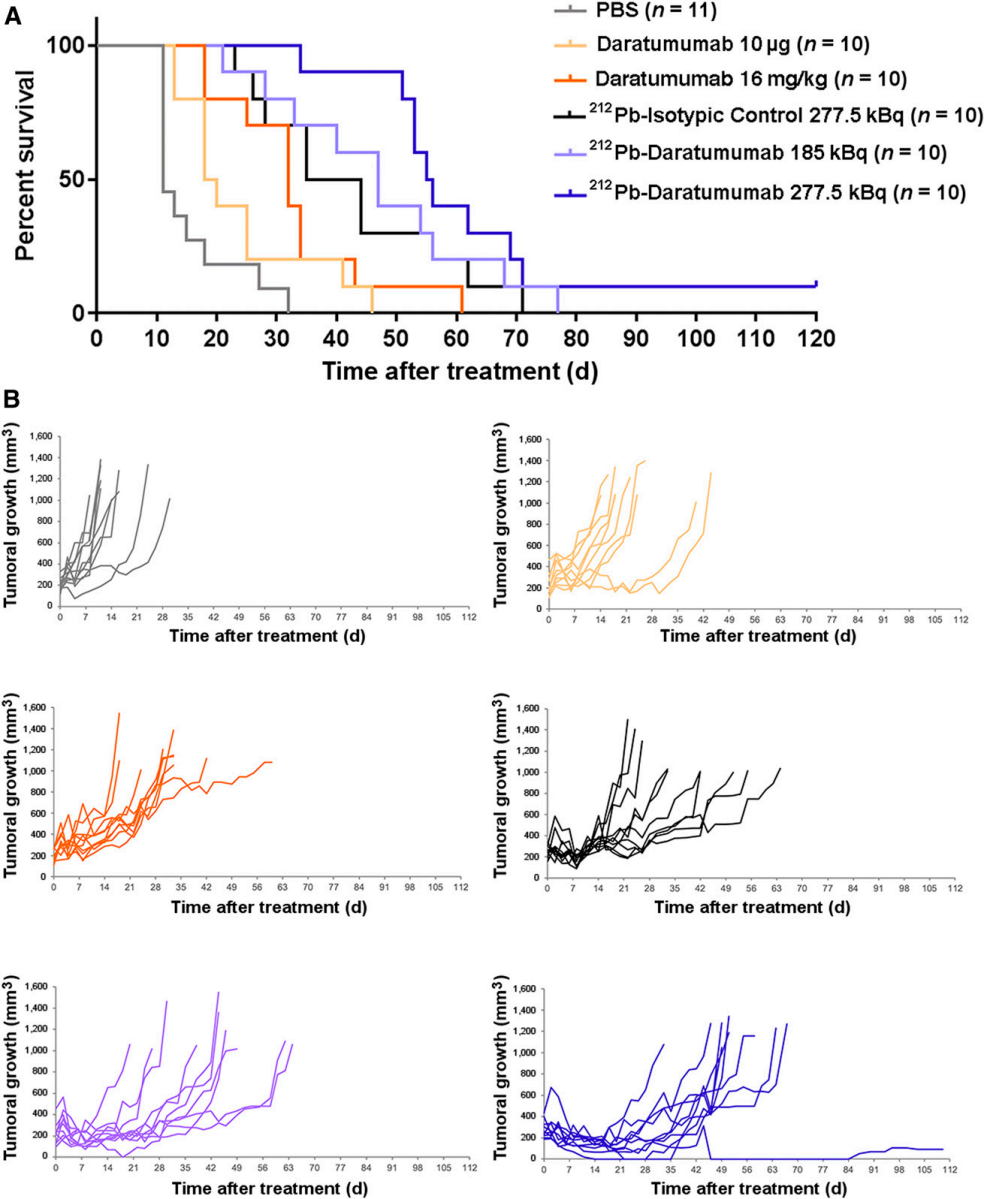
Efficacy of ^212^Pb-daratumumab treatment on Rag2^−/−^γC^−/−^ bearing subcutaneous xenograft of MM cells. Thirteen days after engraftment, mice received PBS, daratumumab (10 μg or 16 mg/kg), ^212^Pb-isotypic control (277.5 kBq), or ^212^Pb-daratumumab (185 or 277.5 kBq). (A) Kaplan–Meier survival analysis. Data were analyzed with log-rank test. (B) Individual tumoral growth evolution. Tumor volume was measured 3 times per week. Mice were euthanized when tumors were ≥1 cm^3^ or when 120 d of survival was reached (end of experiment).

Efficacy studies were conducted on Rag2^−/−^γC^−/−^ bearing subcutaneous xenografts of RPMI8226 MM cells. Survival curves and tumoral growth after treatment are presented in Figure 7. In the control group (PBS), a median survival of 11 d was observed (Fig. 7A). All treatments except daratumumab at 10 μg (equivalent amount used for the radiolabeled antibody) induced a significant median survival time increase (*P <* 0.001): 32, 39.5, 47, and 55.5 d for daratumumab at 16 mg/kg (dose used in clinical practice), ^212^Pb-isotypic control at 277.5 kBq, and ^212^Pb-daratumumab at 185 kBq and 277.5 kBq, respectively. Nevertheless, the survival increase induced by ^212^Pb-daratumumab at 277.5 kBq was significantly higher than that with cold daratumumab (*P <* 0.001). ^212^Pb-daratumumab at 277.5 kBq induced a tumoral regression from day 4 to day 25 after treatment, but a resumption of tumoral growth was then observed except for 1 mouse (Fig. 7B).

## DISCUSSION

Recent advances in MM therapies, such as immunomodulatory drugs and proteasome inhibitors, have significantly prolonged the survival of MM patients over the last decade. However, the prognosis for patients with relapsed MM remains poor. RIT is a therapeutic modality that is not cross-resistant with chemotherapy or other therapeutic agents. This strategy has already shown its value in hematologic cancers, but there are few studies on RIT in MM.

Chérel et al. have studied RIT efficiency in MM with ^213^Bi-anti-CD138 and showed significant efficacy in murine myeloma models (15). In our study, we targeted CD38 because its expression is high and uniform on malignant plasma cells but relatively low on normal lymphoid and myeloid cells and on nonhematopoietic tissues (16). Recent studies confirmed the pertinence of targeting CD38 in the treatment of MM (17*,*^18^).

Most MM RIT studies have used α-emitters. In fact, because MM cells are found either isolated in bone marrow or in small clusters, α-particles have a theoretic advantage over β-particles because of their high-linear-energy transfer and shorter range of action. Cell destruction would be more selective and irradiation less harmful to adjacent cells (19). As anticipated, a study of an anti-CD138 coupled with either ^213^Bi or ^177^Lu revealed the advantages of α-RIT over β-RIT in the treatment of MM in a preclinical model (20). Unfortunately, the fact that ^213^Bi is a short-lived α-emitter (45 min) hampers its use in therapy. We chose to evaluate the α-emitter ^212^Pb, selected for its longer half-life (10.6 h), which is more suitable with regard to antibody kinetics.

In this study, in vitro experiments showed ^212^Pb-daratumumab–specific effects on proliferation of cells with high CD38 expression, as is consistent with previous observations by Teiluf et al. on different MM cell lines, including RPMI8226. Treatment with ^213^Bi-anti-CD38 for 48 h induced a 50% lethal dose of 185 kBq/mL (21). In our experiment, the 50% lethal dose was 0.370 kBq/mL with 4 d of incubation. The high superior efficacy of ^212^Pb when compared with ^213^Bi correlates with its longer half-life as observed by Milenic et al., who compared the effects of ^212^Pb and ^213^Bi on human carcinoma cell line growth: concentrations needed to reach the same level of efficiency were 30–40 times lower for ^212^Pb than for ^213^Bi (22).

^212^Pb-daratumumab biodistributions in mice bearing CD38-positive tumors are consistent with prior published data on ^89^Zr-labeled daratumumab (23*,*^24^). We observed a specific tumor uptake that rapidly increased with time after injection, to reach 15 %ID/g at 18 h and 20–25 %ID/g at 48 h. Imaging data underlined a peripheral tumor fixation on large tumors, suggesting that small lesions might be the best indication for ^212^Pb-RIT or RIT in general (our first idea) because of the slow tumor penetration of full-length antibodies in large tumors. Some uptake was observed in liver, spleen, kidney, and lung (∼10 %ID/g), whereas uptake was minimal in other healthy tissues. Blood activity rapidly decreased and was around 20 %ID/g after 18 h.

Because of the low ^212^Pb activity injected and the detection sensitivity, whole-body SPECT/CT images using ^212^Pb-mAbs could not be acquired and were therefore performed with ^203^Pb. This radioelement allowed us to consider a theranostic approach for ^212^Pb α-therapy with a chemically identical radiometal, preventing the need for a radionuclide with different physical–chemical properties that would likely result in different pharmacokinetics. Imaging with ^203^Pb could provide an effective approach to optimize therapeutic doses using patient-specific dosimetry calculations and to monitor patient response to targeted radionuclide therapy with ^212^Pb.

Because daratumumab does not bind to mCD38, mCD38 biodistribution and toxicity studies were evaluated with a specific anti-mCD38 mAb. Human CD38 and mouse CD38 share sequence homology (70%) but display a different expression pattern, particularly among lymphocyte subsets and within the B-lineage (25). Murine CD38 is expressed abundantly by all murine B-lineage cells; by contrast, human CD38 is expressed highly by germinal center human B cells and less intensely by other human B-lineage cells. This expression pattern explains the high spleen uptake of ^212^Pb-anti-mCD38 in mice. ^212^Pb-anti-mCD38 biodistribution studies predicted the spleen and liver to be the dose-limiting normal organs; however, the differences in expression patterns limit elemental transposition to human ^212^Pb-daratumumab toxicity.

Hematologic toxicity was studied through blood cell counts and flow cytometry on lymphoid organs after death. We observed a reversible hematologic toxicity similar to that observed by Boudousq et al. with ^212^Pb-trastuzumab and ^212^Pb-irrelevant mAb in intraperitoneal tumor xenograft–bearing nude mice (26). In parallel, we investigated toxicity in the liver and kidneys using a chemical approach. Consistent with the studies of Chérel et al. on ^213^Bi-anti-mCD138, we observed no variations in liver or kidney enzymes in our acute toxicity study (15).

No toxicity was observed with ^212^Pb-daratumumab up to 370 kBq because of the lack of cross-reactivity with mCD38. Nevertheless, considering the ^212^Pb-anti-mCD38 toxicity outcome, for this first efficacy study we chose to test an activity of 277.5 kBq to remain under toxic ^212^Pb-anti-mCD38 activities (370 kBq). In clinical application, injection of cold mAb before RIT could be an attractive option to optimize the therapeutic effect and reduce toxicity.

Efficacy studies were conducted on Rag2^−/−^γC^−/−^ mice bearing RPMI8226 cell-line xenografts. In vitro results warranted the use of this cell line as a relevant xenograft model for in vivo studies. Treatment with ^212^Pb-mAbs significantly reduced tumor growth and prolonged median survival compared with cold daratumumab or PBS. A significant partial efficacy was obtained with ^212^Pb-isotypic control (277.5 kBq), corresponding to the effect of nontargeted RIT, likely because of the enhanced permeability and retention effect in tumors, observed in various studies with ^212^Pb or other radioelements (27). In a single treatment regimen on a relatively high tumor volume at treatment time, the significantly greater inhibition of tumor growth observed with ^212^Pb-daratumumab is encouraging, especially considering the relatively low activities used compared with other studies (27).

## CONCLUSION

These promising results highlight the potency of ^212^Pb-daratumumab in the treatment of MM. The ^212^Pb half-life of 10.6 h, its central production, and its worldwide distribution provide clinical feasibility. To further optimize the effectiveness of ^212^Pb-daratumumab, a fractionated regimen could be tested to improve the long-term efficacy of the therapy and prevent tumoral recurrence.

## DISCLOSURE

This research was supported by BPI France. Amal Saidi is an Orano Med employee. No other potential conflict of interest relevant to this article was reported.

**KEY POINTS**

**QUESTION:** What is the potential of ^212^Pb-daratumumab in the treatment of plasma cell malignancies?
**PERTINENT FINDINGS:** In a single treatment regimen, ^212^Pb-daratumumab induced significant tumor growth inhibition.
**IMPLICATIONS FOR PATIENT CARE:** ^212^Pb-daratumumab could be promising in MM treatment.
