# Supplementary material for: 212Pb α-Radioimmunotherapy Targeting CD38 in Multiple Myeloma: A Preclinical Study
Source: J Nucl Med. 2020 Jul;61(7):1058–65. doi: 10.2967/jnumed.119.239491 (PMC7383085; doi:10.2967/jnumed.119.239491)
Supplement: Supplementary file 1 [file jnm239491SupplementalData.pdf]

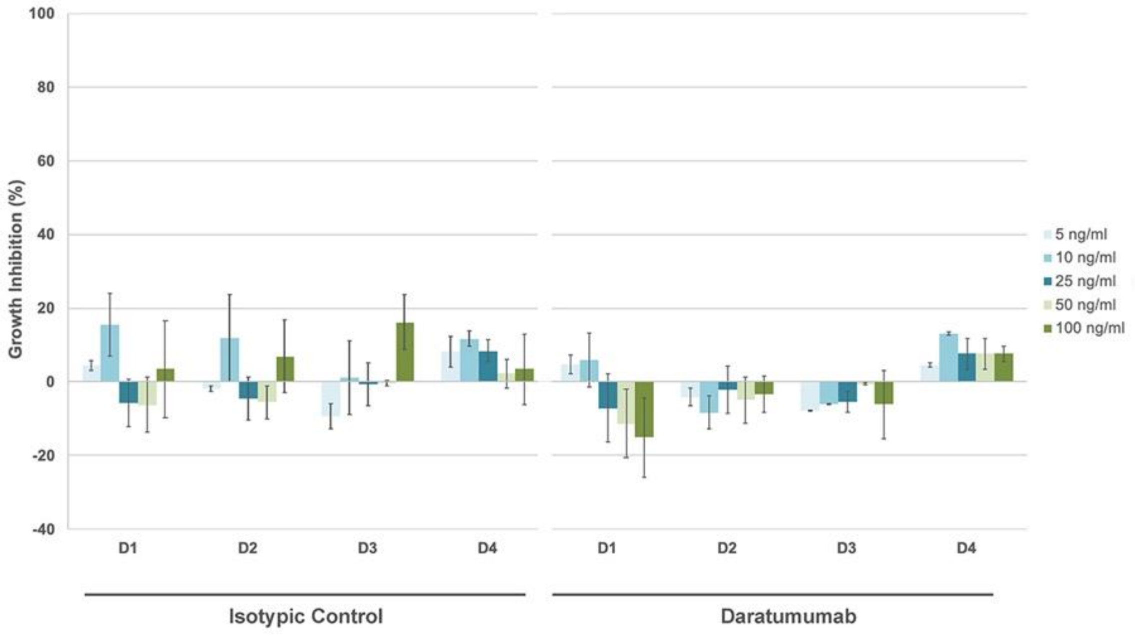

Supplemental FIGURE 1: Percentage of growth inhibition for increasing concentrations of cold-MAb.

Inhibition of cell proliferation, after incubation of RPMI8226 cell line with increasing concentrations of Isotypic Control or Daratumumab (5-100 ng/ml), was measured daily during 4 days by an MTS assay. The growth inhibition percent was calculated, from Day 1 to Day 4, using untreated cells as control on the same day. Data are expressed as % growth inhibition $((1 - DO_{treatment}/DO_{control}) * 100) \pm$ SEM. No significant effect was observed.

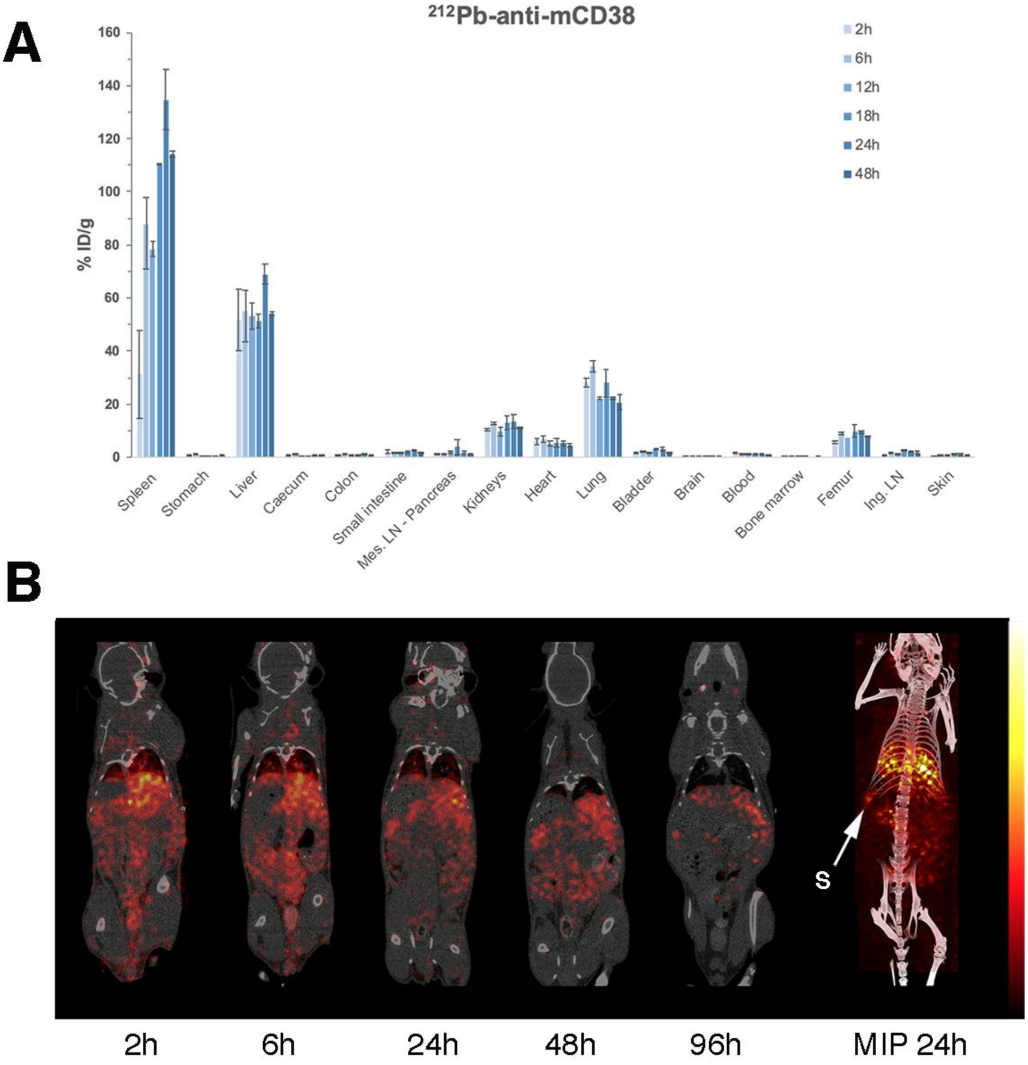

Supplemental FIGURE 2: Biodistribution of radiolabelled anti-mCD38 on Rag2$^{-/-}$γC$^{-/-}$ healthy mice. A: for *post-mortem* biodistribution studies, mice were injected with 185 kBq of ^{212}Pb-anti-mCD38. Groups of 2 mice were euthanized 2, 6, 12, 18, 24 or 48 hours after injection. The radioactivity in normal tissues and blood were quantified by gamma counting, corrected for decay, and expressed as the % ID/g of tissue. B: for *in vivo* studies, mice were imaging, by microSPECT-CT, 2, 6, 24, 48 and 96 hours post-injection of ^{203}Pb-anti-mCD38 (7.4 MBq). MIP: Maximum Intensity Projection; S: Spleen.

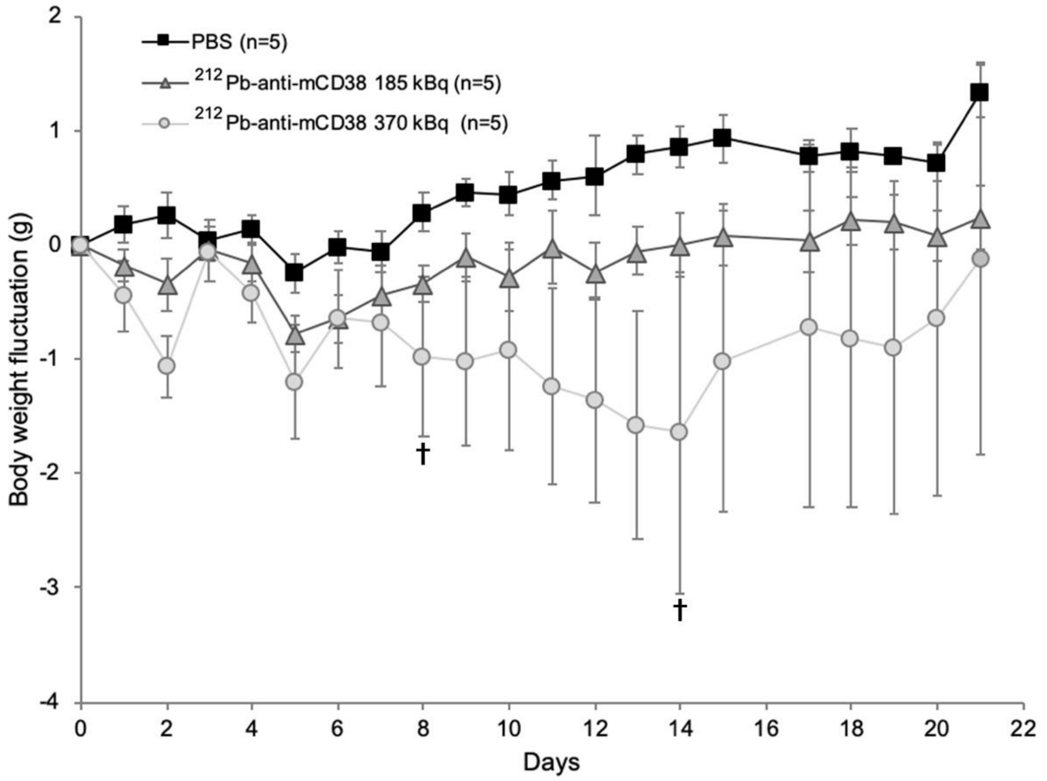

Supplemental FIGURE 3: Body weight variations of C57Bl/6 mice after acute toxicity study.

Mice were injected intravenously with PBS, 185 or 370 kBq of ^{212}Pb-anti-mCD38. Variations of body weight reported relative to Day 0 were represented. Crosses (†): mice euthanized.

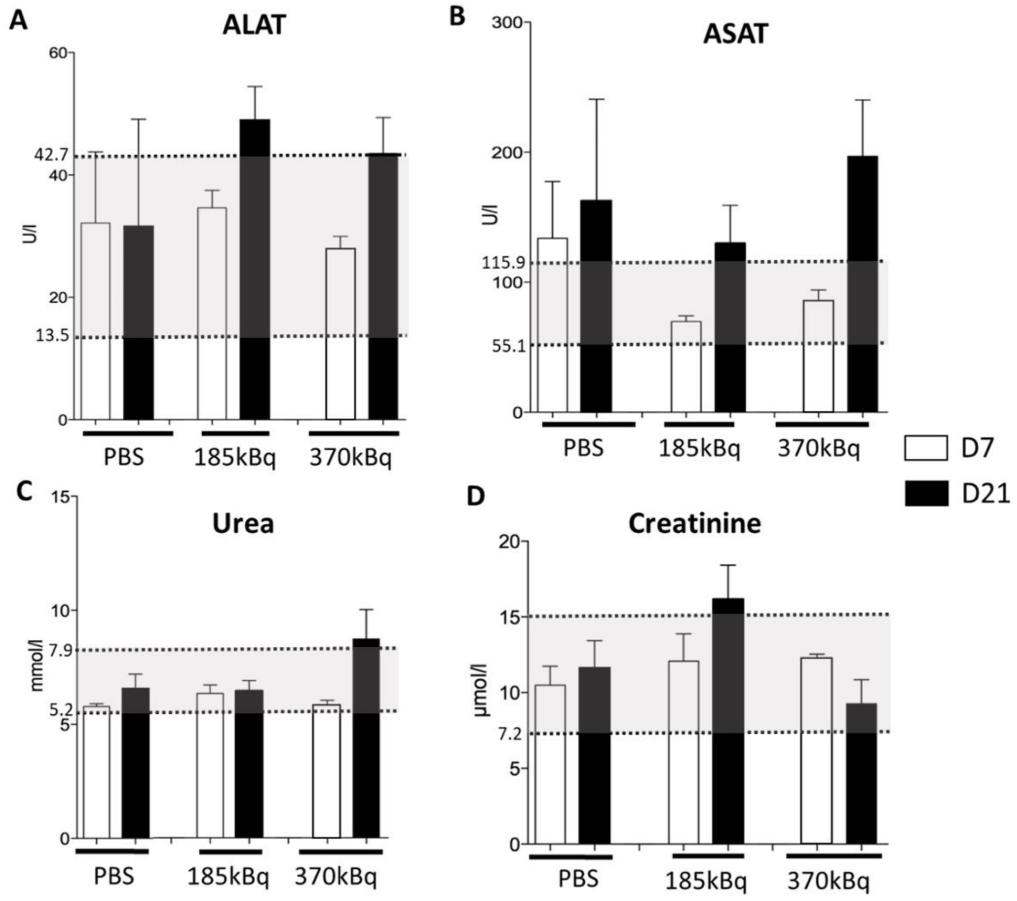

Supplemental FIGURE 4: Effect of acute toxicity on biochemical parameters. C57Bl/6 mice were injected intravenously with PBS, 185 or 370 kBq of ^{212}Pb-anti-mCD38. ALAT (A), ASAT (B), urea (C) and creatinine (D) were measured in blood plasma 7 or 21 days post-injection.
